# Comprehensive Investigation Illustrates the Role of M2 Macrophages and Its Related Genes in Pancreatic Cancer

**DOI:** 10.3390/medicina59040717

**Published:** 2023-04-06

**Authors:** Danying Zhang, Wenqing Tang, Xizhong Shen, Shuqiang Weng, Ling Dong

**Affiliations:** Department of Gastroenterology and Hepatology, Shanghai Institute of Liver Diseases, Zhongshan Hospital, Fudan University, Shanghai 200032, China

**Keywords:** pancreatic cancer, macrophages, TMIGD3, microenvironment, prognosis

## Abstract

*Background and Objectives:* M2 macrophages play an important role in cancers. Our study aimed to illustrate the effect of M2 macrophages in pancreatic cancer (PC). *Materials and Methods:* The open-access data used for analysis were downloaded from The Cancer Genome Atlas Program database, as well as some online databases. R software was mainly used for data analysis based on the specific packages. *Results:* Here, we comprehensively investigated the role of M2 macrophages and their related genes in PC. We performed the biological enrichment of M2 macrophages in PC. Meanwhile, we identified adenosine A3 receptor (TMIGD3) as the interest gene for further analysis. The single-cell analysis showed that was mainly expressed in the Mono/Macro cells based on the data from multiple data cohorts. Biological investigation showed that TMIGD3 was primarily enriched in angiogenesis, pancreas-beta cells and TGF-beta signaling. Tumor microenvironment analysis indicated that TMIGD3 was positively correlated with monocyte_MCPCOUNTER, NK cell_MCPCOUNTER, macrophages M2_CIBERSORT, macrophage_EPIC, neutrophil_TIMER and endothelial cell_MCPCOUNTER. Interestingly, we determined that all the immune functions quantified by single sample gene set enrichment analysis algorithms were activated in the patients with high TMIGD3 expression. *Conclusions*: Our results provide a novel direction for the research focused on the M2 macrophages in PC. Meanwhile, TMIGD3 was identified as an M2 macrophage-related biomarker for PC.

## 1. Introduction

Pancreatic cancer (PC), known as the “king of cancers”, is a highly malignant tumor of the digestive system, about 90% of which manifests in ductal adenocarcinomas with glandular epithelial origin [1]. Statistically, more than 490,000 new cases of PC are diagnosed each year worldwide [2]. Due to the highly insidious and aggressive biology of PC, most patients have no obvious symptoms in the early stage and are in the advanced stage once diagnosed [3]. Therefore, the prognosis for pancreatic cancer is poor, and despite current advances in chemotherapy, targeted therapy, and immunotherapy, the five-year survival rate for PC patients is less than 10% [4]. Therefore, it is still necessary to develop appropriate treatments to improve the survival rate of PC patients.

Macrophages, which are differentiated from monocytes in the blood after they penetrate the blood vessels, are a class of immune cells with phagocytic effects. Macrophages can be divided into two activation types, M1 and M2; M1 mainly secretes pro-inflammatory factors and assumes an important role in the early stage of inflammation, while M2 expresses inhibitory inflammatory factors and plays a role in suppressing the inflammatory response and tissue repair [5]. Since M2 macrophages have unique anti-inflammatory effects, research on M2 macrophages in tumors has become a popular direction. In gastric and breast cancers, M2 macrophages recruited by tumor cells can promote tumor cell metastasis by secreting the Chitinase 3-like 1protein [6]. M2 macrophages can also promote hepatocellular carcinoma progression through the miR-149-5p/matrix metallopeptidase 9 regulatory axis [7]. In renal clear cell carcinoma, M2 macrophages can enhance tumor cell proliferation, invasion and epithelial–mesenchymal transition by secreting CXCL13 [8]. In addition, M2 macrophages were associated with ovarian cancer chemoresistance, glioma department and systemic immunosuppression [9,10]. These studies indicated that M2 macrophages are involved in multiple biological processes in tumors and are a promising therapeutic target. However, there are few studies on M2 macrophages in the PC, and this study will focus on the therapeutic potential of M2 macrophages in the PC.

In this study, we comprehensively investigated the role of M2 macrophages and its related genes in PC. We performed the biological enrichment of M2 macrophages in PC. Meanwhile, we identified the adenosine A3 receptor (TMIGD3) as the interest gene for further analysis. The single-cell expression pattern, biological investigation and tumor microenvironment of TMIGD3 exerting in PC were also explored, making it an underlying biomarker for PC.

## 2. Methods

### 2.1. Acquisition and Pre-Processing of Open-Access Data

In the next step, clinical and mutation data of PC patients were downloaded from The Cancer Genome Atlas (TCGA) database (TCGA-PAAD; file type of “transcriptome profiling” = “STAR − Counts”; file type of clinical information = “bcr xml”). The R codes developed by the authors were used for data consolidation and sorting. Specifically, the individual expression profile file obtained from the TCGA-PAAD project was the “STAR-Counts” form, and it was converted into the “Transcripts per million (TPM)” form. Perl codes developed by the authors were applied to extract clinical information. Before the analysis, the data were pre-processed using the limma package (developed by Ritchie et al.) and the process included probe annotation, data standardization, missing value completion and log2 conversion [11].

### 2.2. Assessment of M2 Macrophages in the PC Microenvironment

CIBERSORT algorithm (developed by Chen et al.) was used to quantify the PC microenvironment based on the gene expression profile, including the relative infiltration level of 22 immune cells and M2 macrophages [12]. Specifically, the support vector regression (SVR) analysis was applied in the CIBERSORT algorithm, which can optimize feature selection and accurately estimate the immune components of tumor biopsy.

### 2.3. Correlation Analysis

Using the correlation analysis, we defined the genes significantly associated with M2 macrophage infiltration as M2 macrophage-related genes, with the potential to affect M2 macrophage polarization in PC microenvironment (|Cor| > 0.3 and *p* < 0.05). The analysis was performed in an R environment and relevant codes were developed by the authors.

### 2.4. Biological Investigation

The evaluation of biological effects was conducted using the Gene Ontology (GO) and Kyoto Encyclopedia of Genes and Genomes (KEGG) analysis, which was performed using the clusterprofiler package (developed by Yu et al.) [13]. The terms with a *p* value < 0.05 were regarded as significant. The species was set as “Human”.

### 2.5. Gene Set Enrichment Analysis (GSEA)

To identify the cancer-related pathway difference between different groups, GSEA analysis was performed based on the Hallmark and KEGG gene set (developed by Subramanian et al.) [14]. Specifically, GSEA can analyze the impact of each gene on specific biological pathways in different subgroups through a predefined set of genes, which can provide more credible conclusions.

### 2.6. Single-Cell Analysis

To evaluate the expression pattern of specific genes at the single-cell level of the PC microenvironment, the project Tumor Immune Single Cell Hub (TISCH) was used (developed by Sun et al.) [15]. TISCH is a large-scale database that integrates single-cell transcriptomic profiles of 76 high-quality tumor datasets across 27 cancer types. The single-cell cohort for analyzed profiles were CRA001160, GSE111672, GSE141017, GSE148673, GSE154763, GSE154778, GSE158356, GSE162708, GSE165399 and GSE176031.

### 2.7. Tumor Microenvironment

Quantification of the tumor microenvironment of PC was conducted using the TIMER (developed by Li et al.), QUANTISEQ (developed by Plattner et al.), CIBERSORT (developed by Chen et al.), XCELL (developed bu Aran et al.) and EPIC algorithms (developed by Racle et al.) [12,16,17,18,19]. The quantification of immune function in PC microenvironment was conducted using the single-sample GSEA (ssGSEA) algorithm (developed by Hänzelmann et al.) [20]. Specifically, the input file was the transcription profile matrix.

### 2.8. Statistical Analysis

The analysis based on the open-access data was conducted using the R software (version 4.0.4). The threshold of statistical significance was set as 0.05. Normally distributed data were analyzed using the Student’s *t*-test. Non-normally distributed data were analyzed using the Mann–Whitney U test. 

## 3. Results

**Figure 1:**
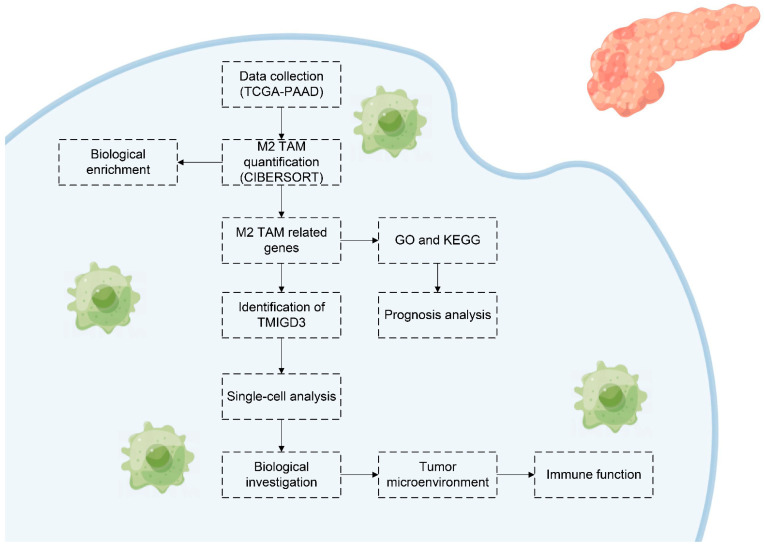
The flow chart of the whole study.

The whole flow chart of our study is shown in Figure 1. In our study, we comprehensively explored the M2 macrophages, as well as the related molecules based on the open-access data. Firstly, we quantified the infiltration level of M2 macrophages through the CIBERSORT algorithm. We explored the biological role M2 macrophages exert and identified the genes significantly associated with M2 macrophage polarization. The TMIGD3 was selected for further analysis, including the single-cell analysis, biological enrichment, tumor microenvironment and immune function analysis.

### 3.1. The Biological Effect of M2 Macrophages in PC

**Figure 2:**
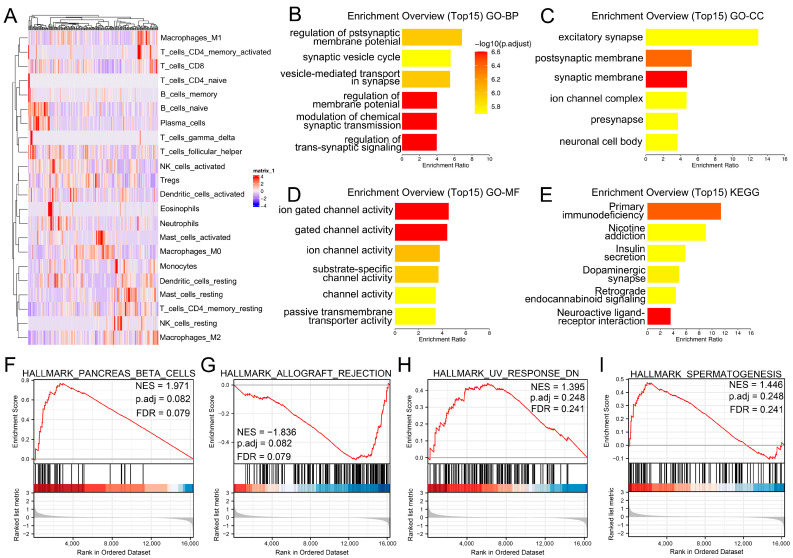
Role of M2 macrophages in PC. Notes: (**A**) CIBERSORT algorithm was used to quantify the PC microenvironment; (**B**) GO-BP analysis of M2 macrophages; (**C**) GO-CC analysis of M2 macrophages; (**D**) GO-MF analysis of M2 macrophages; (**E**) KEGG analysis of M2 macrophages; (**F**–**I**) Hallmark analysis of the M2 macrophages (the red color under the black line represents the gene has a promoting effect on the activity of this pathway, while blue color has the opposite effect).

Based on the CIBERSORT algorithm, the infiltration level of M2 macrophages in the PC microenvironment was quantified (Figure 2A). Enrichment analysis of GO Biological Process (BP) showed that the top six enriched terms M2 macrophages involved in were regulation of postsynaptic membrane potential, synaptic vesicle cycle, vesicle-mediated transport in the synapse, regulation of membrane potential, modulation of chemical synaptic transmission and regulation of trans-synaptic signaling (Figure 2B); the top six enriched terms of GO Cellular Component (CC) were excitatory synapse, postsynaptic membrane, synaptic membrane, ion channel complex, presynapse and neuronal cell body (Figure 2C); the top six enriched terms of GO Molecular Function (MF) were ion-gated channel activity, gated channel activity, ion channel activity, substrate-specific channel activity, channel activity and passive transmembrane transporter activity (Figure 2D); the top six enriched terms of KEGG were primary immunodeficiency, nicotine addition, insulin secretion, dopaminergic synapse, retrograde endocannabinoid signaling and neuroactive ligand–receptor interaction (Figure 2E). GSEA analysis based on Hallmark indicated pancreas beta cells, allograft rejection, UV response DN and spermatogenesis (Figure 2F).

### 3.2. Identification of the M2 Macrophages-Related Genes

**Figure 3:**
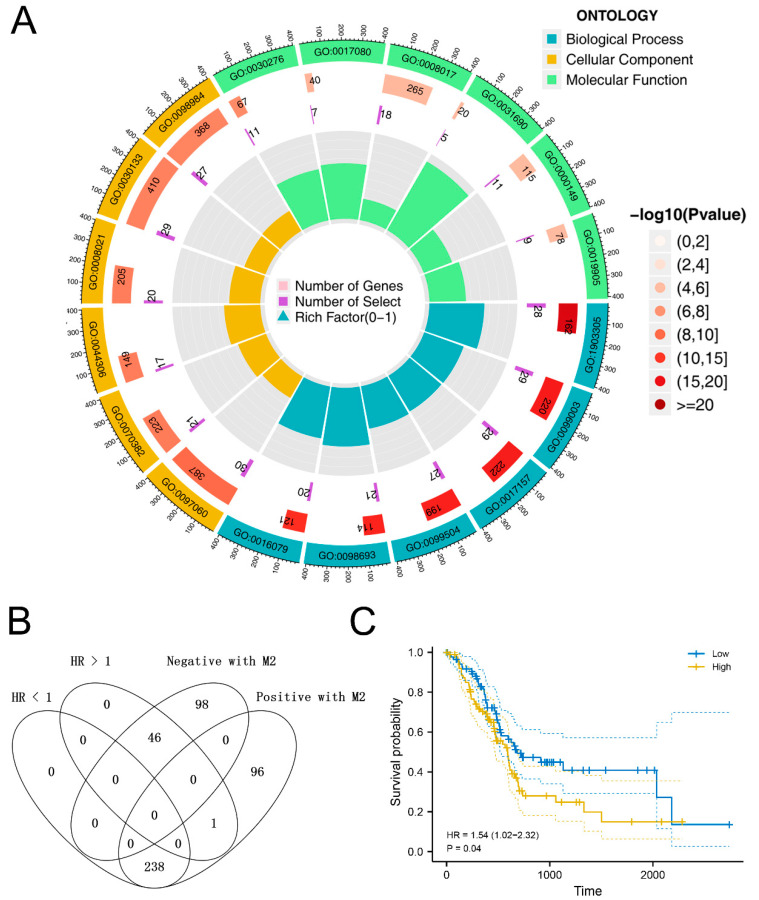
Identification of the M2 macrophage-related genes. Notes: (**A**) GO and KEGG analysis of M2 macrophages-related genes; (**B**) TMIGD3 was positively correlated with M2 macrophages and also a risk factor for PC; (**C**) KM survival curves of TMIGD3 in PC.

Based on the correlation analysis mentioned above, we identified 479 genes significantly correlated with the M2 macrophage polarization (Figure S1). GO and KEGG analysis revealed that these genes were primarily enriched in GO:0017080, GO:0008017, GO:0031690, GO:0000149, GO:0019905, GO:1903305, GO:0099003, GO:0017157, GO:0099504, GO:0098693, GO:0016079, GO:0097060, GO:0070382, GO:0044306, GO:0008021, GO:0030133, GO:0098984 and GO:0030276 (Figure 3A). Finally, we performed the univariate Cox regression analysis to select the molecules significantly associated with patient prognosis. Considering the cancer-promoting effect of M2 macrophages in PC from previous studies, we intersected the molecules positively correlated with M2 macrophages and risk factors, as well as the molecule negatively correlated with M2 macrophages and protective factors (Figure 3B). Then, we tried to identify the genes meeting the following conditions: 1. HR > 1 and positively correlated with M2 macrophages; 2. HR < 1 and negatively correlated with M2 macrophages. Only TMIGD3 was noted for its positive correlation with M2 macrophages and its risk factor characteristic (Figure 3C). TMIGD3, also named ADORA3, is a member of the adenosine receptor family, which is involved in many intracellular signaling pathways and physiological functions. However, its presence has not been reported in pancreatic cancers, as well as in the aspect of M2 macrophages. Therefore, we selected TMIGD3 for further analysis.

### 3.3. Expression Pattern of TMIGD3 in PC

**Figure 4:**
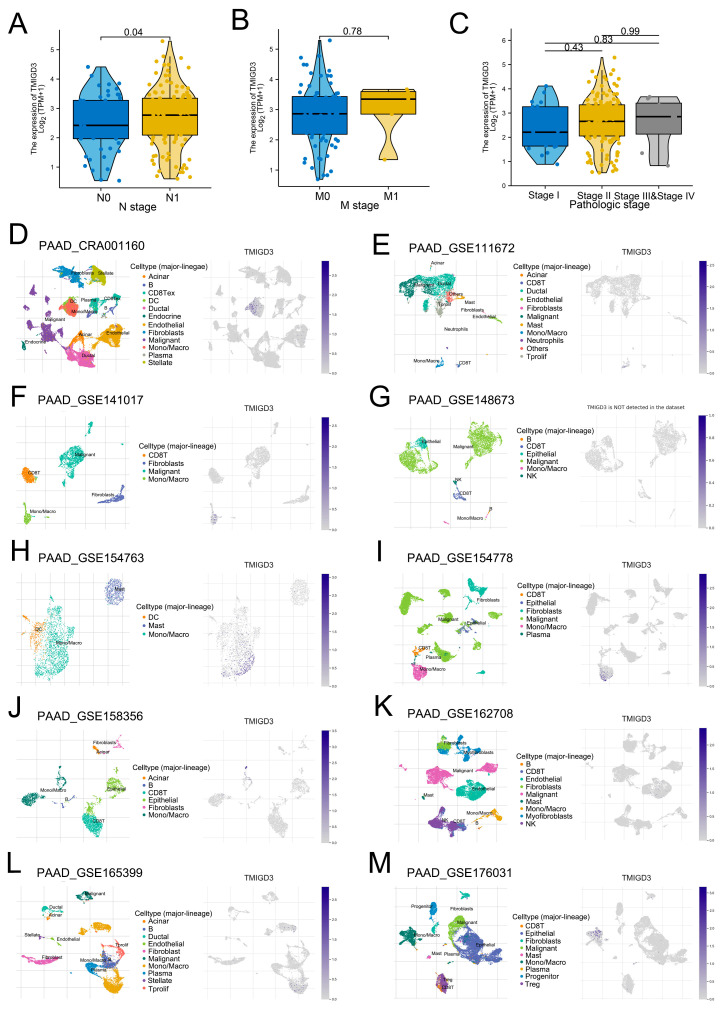
Expression pattern of TMIGD3 in PC. Notes: (**A**–**C**) Expression level of TMIGD3 in patients with different clinical features; (**D**–**M**) The single-cell gene of TMIGD3 in PC.

Kaplan–Meier (KM) survival curves showed that the patients with high TMIGD3 level might have worse survival performance (Figure 3C and Figure S2). Following this, we evaluated the expression level of TMIGD3 in patients with different clinical features, including N classification, M classification and clinical stage. Although not statistically significant, we observed a significantly high level of TMIGD3 in patients with worse clinical features (Figure 4A–C). Single-cell analysis revealed that TMIGD3 was mainly expressed in the Mono/Macro cells based on the data from CRA001160, GSE111672, GSE141017, GSE148673, GSE154763, GSE154778, GSE158356, GSE162708, GSE165399 and GSE176031 (Figure 4D–M and Figure S3). 

### 3.4. Biological Role of TMIGD3 in PC

**Figure 5:**
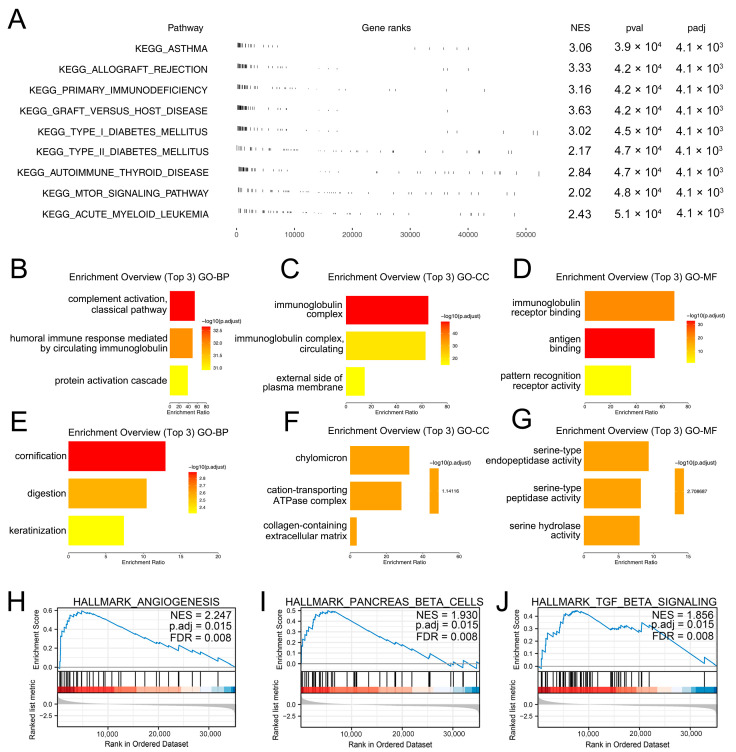
The biological role of TMIGD3. Notes: (**A**) GSEA analysis based on the KEGG gene set; (**B**–**G**) GO analysis of TMIGD3; (**H**–**J**) GSEA analysis based on Hallmark gene set (the red color under the black line represents the gene has a promoting effect on the activity of this pathway, while blue color has the opposite effect).

**Figure 6:**
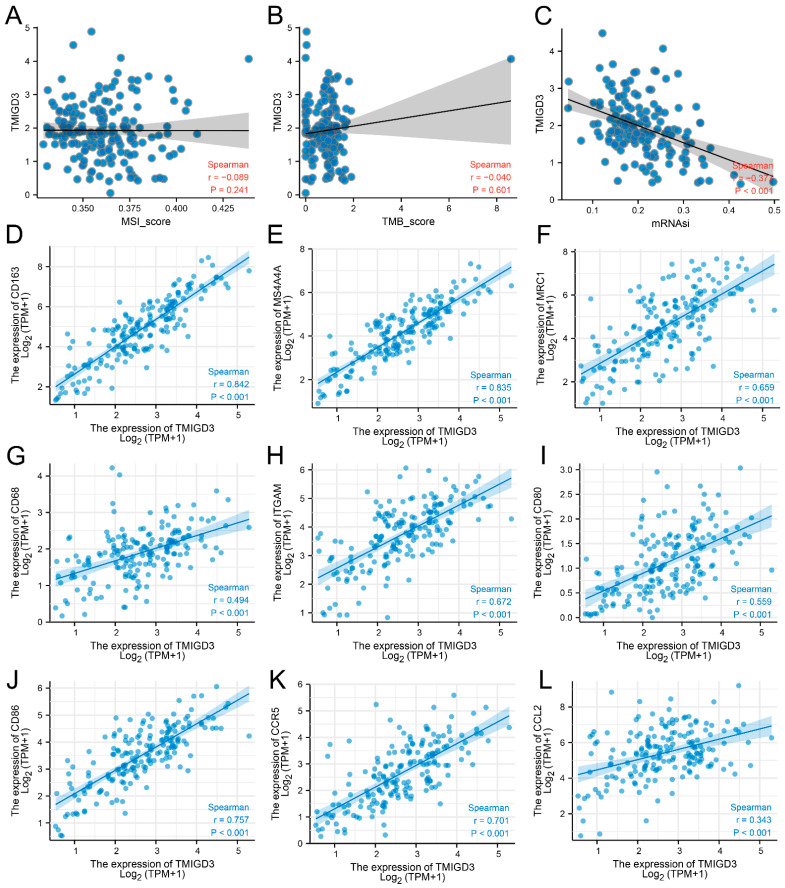
TMIGD3 is associated with the marker of macrophages. Notes: (**A**–**C**) Correlation between the TMIGD3 and TMB, MSI and mRNAsi; (**D**–**L**) Correlation between the TMIGD3 and markers of macrophages.

Subsequently, we tried to investigate the potential biological role of TMIGD3 in PC. GSEA analysis indicated that the TMIGD3 was primarily enriched in asthma, allograft rejection, primary immunodeficiency and graft versus host disease (Figure 5A). In addition, we discovered that the TMIGD3 was positively correlated with the activity of complement activation, classical pathway, humoral immune response mediated by circulating immunoglobulin, protein activation cascade (Figure 5B, GO-BP), immunoglobulin complex, immunoglobulin complex, circulating, external side of the plasma membrane (Figure 5C, GO-CC), immunoglobulin receptor binding, antigen binding and pattern recognition receptor activity (Figure 5D, GO-MF), while it was negatively correlated with cornification, digestion, keratinization (Figure 5E, GO-BP), chylomicron, cation-transporting ATPase complex, collagen-containing extracellular matrix (Figure 5F, GO-CC), serine-type endopeptidase activity, serine-type peptidase activity and serine hydrolase activity (Figure 5G, GO-MF). GSEA analysis based on the Hallmark gene set indicated that TMIGD3 was enriched in angiogenesis, pancreas-beta cells and TGF-beta signaling (Figure 5H–J). We next evaluated the correlation analysis between TMIGD3 and tumor mutational burden (TMB), microsatellite instability (MSI) and mRNAsi score. Results showed that TMIGD3 had no significant effect on TMB and MSI, but was negatively correlated with the tumor stemness index, mRNAsi (Figure 6A–C). Moreover, we discovered that the TMIGD3 was positively correlated with the markers of M2 macrophages (Figure 6D–F, CD163, membrane-spanning 4-domains, subfamily A, member 4A, mannose receptor, C type 1), macrophages (Figure 6G–H, CD68, integrin, alpha M), and cancer-associated macrophages (Figure 6I–L, CD80, CD86, chemokine receptor 5 and chemokine ligand 2).

### 3.5. Effect of TMIGD3 on PC Microenvironment

**Figure 7:**
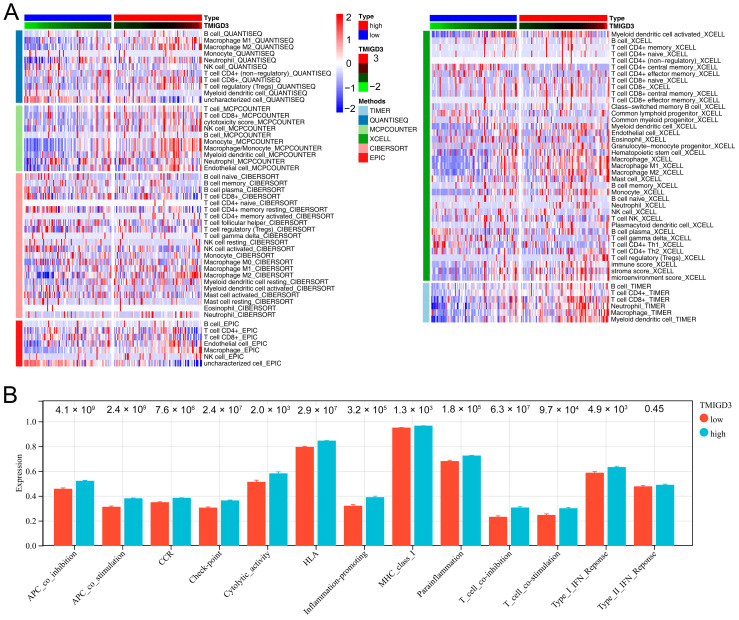
Effect of TMIGD3 on the tumor microenvironment. Notes: (**A**) Effect of TMIGD3 on the cells in tumor microenvironment quantified by multiple algorithms; (**B**) Effect of TMIGD3 on immune function.

We next explored the effect of TMIGD3 on the PC microenvironment. Based on the quantified results, we determined that the TMIGD3 was positively correlated with monocyte_MCPCOUNTER, NK cell_MCPCOUNTER, macrophages M2_CIBERSORT, macrophage_EPIC, neutrophil_TIMER and endothelial cell_MCPCOUNTER (Figure 7A). Interestingly, we discovered that all the immune functions quantified by ssGSEA algorithms were activated in the patients with high TMIGD3 expression, including APC_co_inhibtion, APC_co_stimulation, CCR, Checkpoint, Cytolytic_activity, HLA, inflammation-promoting, MHC_class_I, parainflammation, T_cell_co-inhibition, T_cell_co-stimulation, Type_I_IFN_Response and Type_II_IFN_Response (Figure 7B).

## 4. Discussion

During the past two decades, there has been a doubled number of pancreatic cancer cases worldwide. A total of 441,000 new cases of pancreatic cancer were reported in the world in 2017 compared to 196,000 cases in 1990 [21,22]. Due to the absence of specific clinical symptoms such as back pain, loss of appetite, loss of weight, etc., pancreatic cancer is often diagnosed at a late stage. Pancreatic cancer poses a high risk of death due to the lack of effective screening, as well as the fact that most patients are over the age of 40 and very few are younger [22]. As a result of 80–85% of patients not being able to accept surgery or having systemic metastases, the five-year survival rate is approximately 10% [23]. Due to improved diagnostic methods and radiotherapy technologies, the five-year survival rate for patients with localized and resectable pancreatic cancer in the early stage has increased to 20% [1]. Despite the advancement of treatment approaches, patients with advanced disease or metastases only see modest improvements in their outcomes. Moreover, no targeted therapy has yet been approved or determined useful for treating pancreatic cancer patients, and medical therapeutic innovations for treating pancreatic cancer have been slower than for lung cancer and melanoma [24]. Researching new strategies for detecting pancreatic cancer patients at an earlier stage and identifying potential biomarkers for targeted therapy are critical to making an impact in the clinical world [25].

As antigen presenters to T cells and as phagocytic cells, macrophages are essential for maintaining tissue homeostasis. Although macrophages are adequate in the tumor microenvironment (TME), they can polarize from the inflammation status (M1-like) to anti-inflammation status (M2-like), providing weak antigen presenters to T cells to help cancer cells escape immune response [26]. Sadly, M2 macrophages are mainly found in TME and show protumor effects through accelerating tumor growth, proliferation, angiogenesis, and invasion [27]. The polarization status of macrophages and their functions are altered when macrophages are induced to TME by cancer cells that secrete a wide range of metabolites, cytokines, and exosomes [28]. Tumor promotion activities can be attributed to macrophage subpopulations since changes in macrophage polarization affect tumor development, progression, and metastasis [29]. It is important to regulate the inflammatory TME and limit the supporting effects of M2 macrophages, such as decreasing the recruitment of M2 macrophages and blocking key cytokines to improve the immunosuppressive TME [28]. Inhibiting tumor development and metastasis by targeting M2 macrophages in the TME would therefore be a novel therapeutic approach.

In our study, we comprehensively investigated the role of M2 macrophages and its related genes in PC. We performed the biological enrichment of M2 macrophages in PC. For the researchers in this aspect, our results can provide indicative meaning for their future studies, including the biological pathways of M2 macrophages in pancreatic cancer, which may be helpful to develop novel targets. Meanwhile, we identified TMIGD3 as the interest gene for further analysis. The single-cell expression pattern, biological investigation and tumor microenvironment of TMIGD3 exerting in PC were also explored, making it an underlying biomarker for PC. Considering the prognosis and immune relevance of TMIGD3, detecting TMIGD3 in clinical environments can indicate the survival performance and M2 macrophage infiltration to some extent, which may help to better assess the patient’s condition and implement targeted treatment measures.

Our result showed that the TMIGD3 was enriched in angiogenesis, pancreas-beta cells and TGF-beta signaling. A recent study revealed that in osteosarcoma cells, TMIGD3 suppresses aggressiveness primarily by inhibiting NF-κB activity [30]. Meanwhile, angiogenesis is widely accepted as an important mechanism by which tumors obtain nutrients and oxygen. Specifically, PC is a fibrotic/hypoxic tumor that contains a large amount of stroma, so the metastasis and prognosis of patients was related to the angiogenesis pathway [31]. It is still noteworthy that some pancreas-beta cells are responsible for the inhibition of PC, which migrates from the islets and forms ductal cancerous tissue [32]. More importantly, as a regulator of tissue renewal, TGF-beta signaling may contribute to the evasion of the PC from immune-mediated elimination [33]. Our study provides evidence that by enriching these pathways, TMIGD3 may have an important role in the carcinogenesis of PC.

For the tumor microenvironment analysis, we discovered that TMIGD3 was positively correlated with monocyte, M2 macrophages and endothelial cell. Among patients with esophageal cancer, TMIGD3 played a key role in the immune/stromal scores, and disease stage pathological type was associated with a shorter overall survival rate [34]. It was determined that monocytes from patients with PC displayed constitutive phosphorylation and a weaker response to stimuli, indicating an abnormal activation and suppression of the immune system [35]. Moreover, in the pancreatic ductal adenocarcinoma (PDAC) microenvironment, the overexpression of M2 polarized macrophages may contribute to both inflammation and immune response, enhancing tumor growth and metastasis [36]. Additionally, the expression level of endothelial cells is significantly associated with PC patient survival [37]. Interestingly, our findings may provide crucial information about the local infiltration of these cells, suggesting that TMIGD3 may act as a chemokine, exerting its effect by influencing these cells in PC.

The rapid development of bioinformatics and open-access data is helpful for researchers [38,39,40,41]. Even though our research identified several characteristics of pancreatic cancer, there were some deficiencies we need to address in future investigations. Firstly, due to the relatively small samples of transcriptomic data and clinical data accessed from GEO and TCGA databases, potential selection bias could exist. To overcome this, external datasets with complete and large clinical information and gene expression data will be needed. Secondly, a large clinical study and experimental validation are also necessary to validate the robustness of our results. Therefore, further studies are required to address the limitations of the present study.

## Data Availability

The data presented in this study are available on request from the corresponding author. The data are not publicly available due to the need of further research.

## Supplementary Materials

The following supporting information can be downloaded at: https://www.mdpi.com/article/10.3390/medicina59040717/s1, Figure S1: The genes significantly with M2 macrophages; Figure S2: Kaplan-Meier survival curves of TMIGD3; Figure S3: Single cell analysis of TMIGD3.
