# Supplementary material for: Comprehensive Investigation Illustrates the Role of M2 Macrophages and Its Related Genes in Pancreatic Cancer
Source: Medicina (Kaunas). 2023 Apr 6;59(4):717. doi: 10.3390/medicina59040717 (PMC10146353; doi:10.3390/medicina59040717)
Supplement: Supplementary file 1 [file medicina-59-00717-s001.zip › medicina-2140249-supplementary.pdf]

Supplementary

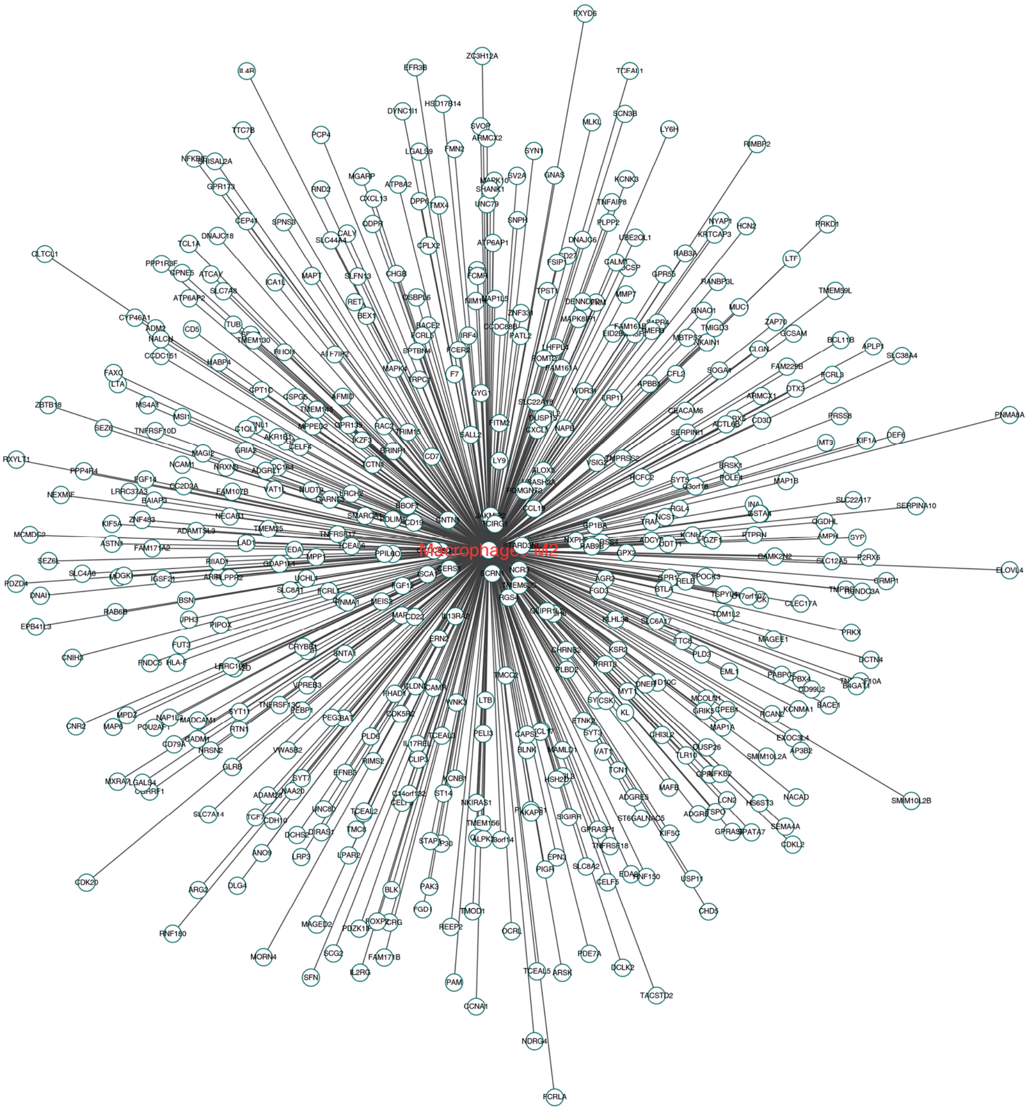

Figure S1. The genes significantly correlated with M2 macrophages

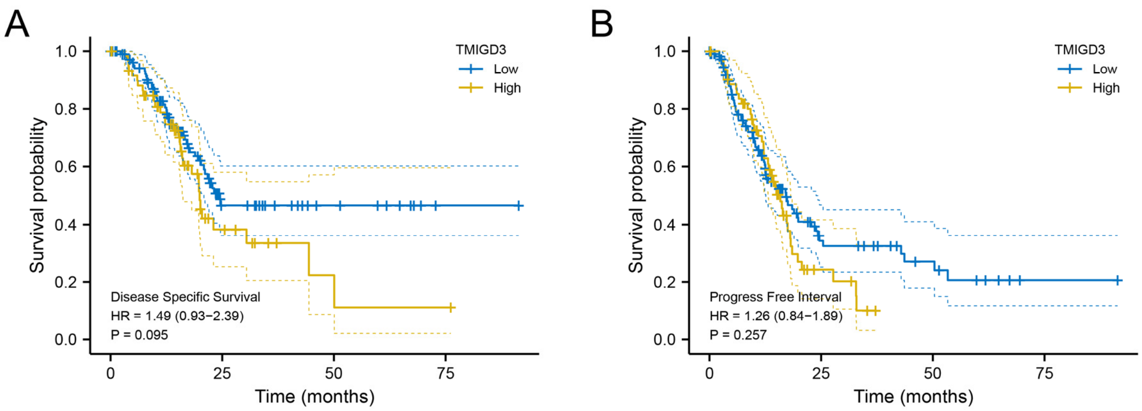

Figure S2. Kaplan-Meier survival curves of TMIGD3

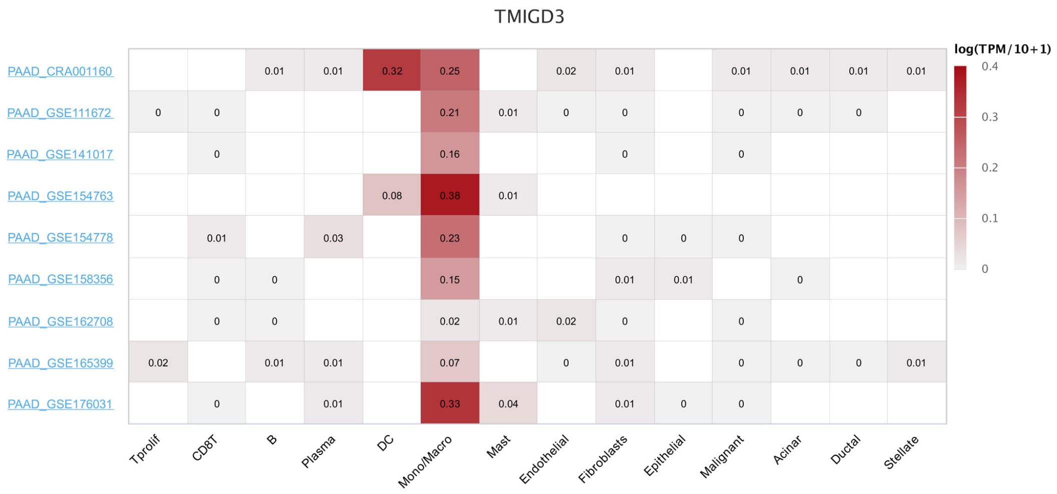

Figure S3. Single-cell analysis of TMIGD3
